# Weight loss among female health care workers- a 1-year workplace based randomized controlled trial in the FINALE-health study

**DOI:** 10.1186/1471-2458-12-625

**Published:** 2012-08-08

**Authors:** Jeanette R Christensen, Kristian Overgaard, Isabella G Carneiro, Andreas Holtermann, Karen Søgaard

**Affiliations:** 1 Department of Sport Science, Aarhus University, Aarhus, Denmark; 2 National Research Centre for the Working Environment, Copenhagen, Denmark; 3 Institute of Sports Science and Clinical Biomechanics, University of Southern Denmark, Odense, Denmark

## Abstract

**Background:**

Weight management constitutes a substantial problem particularly among groups of low socio-economic status. Interventions at work places may be a solution, but high quality worksite interventions documenting prolonged weight loss are lacking. This paper presents results of an intervention aimed to achieve a 12 months weight loss among overweight health care workers.

**Methods:**

Ninety-eight overweight female health care workers were randomized into an intervention or a reference group. The intervention consisted of diet, physical exercise and cognitive behavioral training during working hours 1 hour/week. The reference group was offered monthly oral presentations. Several anthropometric measures, blood pressure, cardiorespiratory fitness, maximal muscle strength, and musculoskeletal pain were measured before and after the 12-months intervention period. Data were analyzed by intention-to-treat analysis.

**Results:**

The intervention group significantly reduced body weight by 6 kg (p < 0.001), BMI by 2.2 (p < 0.001) and body fat percentage by 2.8 (p < 0.001). There were no statistical reductions in the control group, resulting in significant differences between the two groups over time.

**Conclusions:**

The intervention generated substantial reductions in body weight, BMI and body fat percentage among overweight female health care workers over 12 months. The positive results support the workplace as an efficient arena for weight loss among overweight females.

**Trial registration:**

NCT01015716.

## Background

Female health care workers have a high prevalence of overweight and obesity [1]. Since excessive bodyweight comprises one of the largest health-related challenges in the western countries, effective initiatives for long-term weight management are needed. People outside the workforce tend not to be as healthy as people in the workforce. However in a preventive perspective (before people drop out of the workforce often because of health-related issues) and because of occupational clustering of health-related issues, workplaces may be an appropriate arena for prevention of chronic disease.

In a recent systematic review it was concluded that workplace interventions aiming at reducing body weight generally confer only modest positive results [2]. However many of these studies targeted white-collar workers in occupational settings where overweight rates were not reported. It is possible that in an occupational setting with a higher prevalence of overweight, the implementation of a weight loss intervention will be more successful. In addition, health care workers generally belong to a lower socioeconomic group [3] than white-collar workers and there is evidence that health campaigns and interventions targeting the general public does not effectively change health behaviors in low socioeconomic groups. Therefore, it is attractive to examine effects of a targeted weight loss intervention at workplaces with a large proportion of overweight female health care workers.

Interventions aiming at weight loss have implemented elements like diet, physical exercise and cognitive behavioral approaches. Recent reviews have suggested an integration of these three elements for increasing the probability of weight loss with long-term maintenance [4,5].

Apart from health problems most commonly related to overweight like diabetes and hypertension, overweight is also associated with reduced productivity and work ability (WAI), aerobic fitness, muscular strength and increased risk for musculoskeletal pain [6-12]. Both physical capacities and musculoskeletal pain are important determinants of work ability and sickness absence of health care workers [13]. Therefore, it is also of interest to investigate if an effective weight-management program may improve physical capacities and musculoskeletal pain.

The main purpose of this study was to investigate effects on body weight of a 12 month workplace intervention consisting of diet, physical exercise and cognitive behavioral training among overweight female health care workers. Additionally, effects on anthropometric variables, blood pressure, muscle strength, aerobic fitness and musculoskeletal pain were evaluated.

## Methods

### Study design

The present study, termed FINALE-health is a cluster randomized single-blinded controlled trial conducted from May 2009 to the end of June 2010. The study consisted of 12 months intervention with tests performed at baseline, after three months and after one year. The intervention and effects after the first three months have previously been reported [14]. The present paper presents results after 12 months of the intervention. The project was ethically approved by The Central Denmark Region Committees on Biomedical Research Ethics (M-20090050), and qualified for registration in the International Standard Randomized Controlled Trial Number Registry (NCT01015716).

### Recruitment and randomization

For detailed description of recruitment and randomization of participants, please see Christensen et al. 2011 [14]. In short, Randers municipality agreed to participate in the study, and one of nine possible areas of the municipality was found eligible to participate. Recruitment of participants was based on the payroll, and all employees were invited to participate in the project. Of 202 invited employees, 158 attended one of three introductory meetings. Among these employees, the predefined target group for the project were those fulfilling the following the inclusion criteria of being female, overweight (i.e. BMI > 25 or body fat % > 33 for age 18–40 or > 34 for age > 40), and being health care worker or primarily working with elderly care. 101 persons fulfilled the inclusion criteria, and 98 consented participation in the study.

All consenters filled out a screening questionnaire [15], were physically tested the following week, and enrolled and were divided in clusters which were randomly allocated to either intervention or reference group. Clusters were created based on information from the screening questionnaire and the management of working teams, day and evening/night shifts and close working relations. This approach was chosen to avoid contamination, and to benefit from the social support in work teams, thereby increasing compliance. The cluster-randomization was balanced on sex, age, job seniority and job type and done by an external research group, with no connection to the workplace or the participants [14].

### Intervention

**Figure 1:**
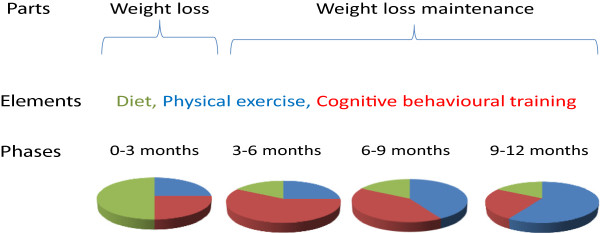
** Overview of the intervention.** The first 3 months consisted of a weight loss period followed by a 9 months weight loss maintenance period. Over the total 12 months intervention, the different elements changed in quantity.

In short, the intervention was carried out one hour a week during work time. The intervention lasted 12 months and consisted of two parts. The first part *(0–3 months)* focused on weight loss and included advice on dietary change based on the Danish Dietary recommendations, calorie counting, weight measurements, weight loss targets, strengthening exercises and initiating leisure time fitness exercise. The second part *(3–12 months)* focused on weight loss maintenance through further intervention with physical exercise and cognitive behavioral training, see Figure 1.

### Dietary intervention

Before the intervention, all participants were encouraged to fill out nine days dietary records [16]. They were used to obtain information on dietary preferences and to create different exemplary courses so food intakes during the weight loss period matched to their normal preferences. Still, to change the participants usual food intake to a healthier diet, the courses were adjusted according to the Danish Dietary recommendations, e.g. reduction of refined sugar, reduction of fat – especially saturated fat, carbohydrates from primarily fiber-rich sources (e.g. whole-grain bread, whole grain pasta etc.), and 600 gram of fruit and vegetables per day. These guidelines are also consistent with new findings regarding weight loss [17]. These courses were proposed for every meal with specific calorie amounts adjusted to suit an individual calorie prescription. In advance, each individual's resting metabolism was calculated, based on gender, age and weight and multiplied by a Physical Activity Level factor (PAL) of 1.8 to estimate the daily energy requirement [18]. 1.000 – 1.200 calories was subtracted from the estimated daily energy requirements and the resulting value was used as individual calorie prescription. The calorie reduction in the present study was set to 1.000 – 1.200 calories to achieve an optimal weight reduction rate of about 1 kg per week [5]. This recommendation is based on a systematic review from 2007 by Franz MJ et al. “Weight-loss outcomes: a systematic review and meta-analysis of weight-loss clinical trials with a minimum 1-year follow-up”. The literature reviewed finds a 1.200 kcal reduction for women and a 1.500 kcal reduction for men in diet and exercise studies [5]. The American Dietetic Association’s Adult Weight Management Evidence-Based Nutrition Practice Guideline supports only a calorie deficit between 500 – 1000 calories [19], thus a calorie deficit between 1.000 – 1.200 calories was chosen”.

During the first three months of the intervention, if weight loss after two weeks was less than expected, the participants had their planned daily calorie intake lowered by further 300 kcal per day. The dietary advices and the weight check occupied approximately 30 min of the weekly session.

During the last nine months of the intervention, every session still began with a weight check. Participants were clearly informed, that the aim of this period was to maintain their weight after the first 3 months loss of body weight, and that they no longer had to continue the reduced daily intake of calories. If participants during the first three months had problems quitting eating unhealthy food such as sweets and biscuits, they were advised to add smaller amounts of these products into their intake. If participants had problems with hunger, they were advised to eat larger and healthy meals. During the last nine months of the intervention, no further dietary counseling was provided unless questions were asked.

### Physical exercise training

During the first 6 months of the intervention, about 15 minutes physical exercise training was included in the weekly session at the workplace (Figure 1). Focus was on strength training to maintain muscle mass in the lower extremities in order to maintain resting metabolism and physical capacity. These exercises were carried out in a meeting room at the workplace and consisted of one and two legged squats, with and without dumb bells and core balls, and lunges walking forward and to each side. Other exercises focused more on general strength, and included exercises for abdominal and back extension, shoulders and arms. Participants brought home a strength training program, picturing these exercises, and were encouraged to perform them twice a week at home. The dose of the instructed physical exercises in the sessions progressed in intensity throughout the weeks of the intervention, by increasing weights and repetitions. In addition to the brief training sessions, participants were encouraged to initiate leisure time aerobic exercises for two hours weekly such as biking, walking, running, swimming or attending different sports in the local area.

From the 6^th^ to the 9^th^ months of the intervention*,* a simple fitness gym was arranged with rubber bands, dumbbells, core-balls, skipping ropes and mattresses plus fitness machines for abdominal and back extension, leg curls and leg press. During this phase, the physical exercise was carried out in the fitness gym as circuit-training, both to progress muscle mass and aerobic fitness. Heart-rate monitors were lent to participants to learn about effective heart rate intensities for increasing aerobic fitness (>70% of maximal heart rate). In this phase, out-door running was also initiated.

During the 9^th^ to the 12^th^ month of the intervention, physical exercises primary took place as different local sports activities. It was the aim that all participants should attend sports at least twice a week (minimum of two hours in total), scheduled in the local area. Each of the seven training teams decided which sport activities to attend together during the sessions, and they ranged from attending fitness centers to zumba, spinning, swimming, water-gymnastics and line dance. Out-door running was still carried out during this phase. For motivation, participants were instructed to monitor leisure time exercises by training log books during the entire year.

### Cognitive behavioral training (CBT)

Before the intervention, a specific CBT training tool based on Linton’s model for coping with chronic pain [20,21] were modified to address discomfort during weight loss and to support a change to a more physically active lifestyle. The cognitive behavioral training tool was based on group discussions and a specifically tailored guideline, containing exercises such as pro-and-con schemes and positive thinking strategies with homework between each session.

During the first three months, about 15 minutes of the weekly sessions was used on CBT (Figure 1), helping the participants to make realistic weight loss targets and find personal strategies to ease hunger. From the 3^th^ to the 9^th^ month of the intervention, about 30 minutes were spent on CBT in the weekly sessions. Focus was to reflect on dysfunctional attitudes and coping behaviors with respect to the weight loss, and discuss functional alternatives and train the implementation of these in everyday life. During the 9^th^ to the 12^th^ months of the intervention*,* about 15 minutes of each session were spent on how to continue healthy behaviors, cope with social contexts and situations involving alcohol, food etc.

### Reference group

The reference group was offered a monthly two-hour oral presentation during working hours. The twelve presentations were based on the Danish National Board of Health and the Ministry of Food, Agriculture and Fisheries public websites and concerned the Danish Dietary recommendations and other health related topics.

### Objective measures

All participants were tested at baseline, after three months and after one year. Each test session lasted one hour and consisted of anthropometrical, health-related and physical capacity measures specified in the following. *Height* was measured without shoes. *Body weight* was measured wearing light clothes, but without socks and shoes. The weight measure was subtracted by 1 kg to compensate for clothing. *Body Fat* was measured by a bio impedance device (TANITA SC-330), which was set to 'standard' as body frame and the participant's age, height and gender were entered. *Waist circumference* was measured over the umbilicus and *hip circumference* on the hip part that gave the greatest circumference. Waist and hip circumference were measured standing up and with clothes on, using an ergonomic circumference measuring tape (Seco 203 Girth measuring tape). *Blood pressure* was measured in seated position after 10 minutes of rest with an electronic blood pressure monitoring device (Artsana CS 410). Three measurements were done one minute apart and the average value was used [22]. Cardiorespiratory fitness was measured using a Monark E327 bicycle ergometer and a heart rate monitor. Participants cycled for five minutes at 70 watts (60 rpm, 1 kp). Within these first five minutes, the test subject was asked to rate their fitness according to the Borg Scale. Hereafter load was increased by 35 watts (1/2 kp) every other minute until the test subject was forced to stop because of exhaustion. An algorithm was used to estimate maximal oxygen uptake (VO_2_-max) [23].

Isometric maximal voluntary strength was obtained with a reproducible standardized setup [24], measuring maximal voluntary handgrip, sitting shoulder elevation, shoulder abduction and back flexion and extension force [25]. The participants performed a minimum of three attempts with steady increasing force to reach maximum within 3–5 seconds. The test was repeated until a maximal of 5 contractions if the last attempt showed a more than 5% increase from the previous maximum. The participant rested at least 30 seconds between each attempt. The maximal attempt was recorded for further analysis. Standardized verbal command and encouragement was given to maximize the effort. *Handgrip* was measured in both hands using a grip strength measurer (La Fayette) [26]. *Shoulder elevation* was measured with a Bofors dynamometer. The subject was seated erect on a chair with legs hanging freely, arms hanging along the side and head facing forward. The distance from pressure points to sternoclavicular joints were measured as the moment arms [27]*. Shoulder abduction* was measured with the Bofors dynamometers with the subject still seated erect on a chair with legs hanging freely, arms along the side and a 90 degrees flexion in the elbows and head facing forward. The distance from pressure points to the lateral epicondyles of the humerus were measured as the moment arms [27]*. Back flexion and extension* were measured with the subject standing, facing/backing a beam and support plate at the spina iliaca anterior superior. The Bofors dynamometer was fixed to pull horizontal with a belt positioned at the vertical level of m. deltoid insertion on the humerus. The distance from the belt to a line through the crista iliaca and lumbalcolumna (L4L5 level) was measured for the moment calculation [28]*.*

Prior to the test session, participants were screened in accordance with the exclusion criteria for the test. The exclusion criteria for one or more of the tests were elevated blood pressure, defined as systolic values higher than 110 mmHg + persons age or diastolic values higher than 100 mmHg regardless of age [22], angina pectoris, heart or lung prescription medication, current or pervious illnesses and trauma, herniated disc, tennis elbow, golf elbow, Carpal Tunnel Syndrome, significant level of musculoskeletal pain at the time of the test, and pregnancy. An example is the bicycle-test. At baseline 35 in the intervention group and 22 in the reference group completed the test, the rest were excluded. After a year, only 23 and 16 in the two groups respectively could complete the re-test. A range of participants in the intervention and the reference group completing the different tests is given in Table 1. The test manager was blinded regarding the participants intervention status, and whenever possible the same test manager tested the subject at all three rounds of tests [14].

**Table 1:** General health, physical capacity and musculoskeletal pain at baseline (test 1) and after 12 months (test 3)

	**Intervention group (n = 54)**			**Reference group (n = 44)**			**P-values**
	**Test 1**	**Test 3**	**P-value**	**Test 1**	**Test 3**	**P-value**	
General health							
Weight (kg)	84.2 (15.9)	78.4 (15.8)	<0.000	83.0 (14.4)	82.7 (14.6)	0.701	<0.000
Body Mass Index (kg/m^2^)	30.7 (5.4)	28.5 (5.5)	<0.000	30.4 (4.9)	30.3 (5.1)	0.792	<0.000
Fat percentage (%)	41.2 (5.7)	38.4 (7.3)	<0.000	40.5 (5.7)	40.4 (6.0)	0.704	<0.000
Waist circumference (cm)	100.1 (13.8)	96.1 (14.9)	0.004	101.6 (12.4)	100.0 (13.4)	0.348	0.030
Hip circumference (cm)	112.4 (11.3)	110.2 (11.8)	0.056	113.3 (10.6)	113.6 (10.9)	0.601	0.026
H/W-Ratio	0.88 (0.08)	0.86 (0.08)	0.002	0.89 (0.07)	0.88 (0.07)	0.097	0.272
Systolic blood pr (mmHg)	134.1 (19.6)	125.5 (15.0)	<0.000	129.3 (11.9)	125.5 (14.9)	0.025	0.094
Diastolic blood pr (mmHg)	85.5 (10.8)	81.7 (9.4)	<0.000	81.7 (8.3)	80.7 (9.2)	0.309	0.109
Physical capacity							
Handgrip Dom si (N)	297.6 (52.4)	304.4 (46.0)	0.112	305.1 (55.3)	302.7 (55.0)	0.266	1.000
R Shoulder elv Nm	73.6 (22.8)	75.8 (20.1)	0.590	62.5 (24.5)	73.4 (26.7)	0.002	0.133
L Shoulder elv Nm	62.0 (22.0)	68.7 (22.2)	0.032	53.8 (24.9)	62.1 (24.8)	0.007	0.858
R Shoulder abd Nm	35.0 (14.5)	45.0 (13.3)	0.001	31.9 (13.2)	46.4 (16.2)	<0.000	0.120
L Shoulder abd Nm	35.5 (12.9)	47.8 (16.0)	<0.000	31.7 (12.6)	46.5 (17.7)	<0.000	0.355
Abdominal Nm	127.0 (32.2)	119.3 (39.3)	0.132	136.0 (54.7)	126.2 (45.5)	0.072	0.974
Back Nm	116.4 (40.4)	138.3 (44.4)	0.004	119.8 (46.4)	134.2 (36.8)	0.021	0.909
VO2 Max	2.06 (0.35)	2.10 (0.40)	0.187	2,13 (0,34)	2.16 (0.44)	0.501	0.543
Physical fitness	25.9 (5.0)	27.5 (6.4)	0.004	26,7 (5,1)	27.5 (5.3)	0.294	0.158
Pain intensity last 7 days (1–10)							
Neck	2.5 (2.5)	1.9 (2.2)	0.050	2.4 (2.6)	2.6 (2.9)	0.659	0.113
Shoulder - right	1.9 (2.7)	1.3 (2.2)	0.049	1.6 (2.4)	1.6 (2.5)	0.841	0.313
Shoulder - left	1.5 (2.3)	1.5 (2.2)	1.000	0.87 (1.7)	1.26 (2.5)	0.207	0.781
Upper back	1.8 (2.3)	1.4 (1.9)	0.096	1.5 (2.6)	1.0 (2.1)	0.190	0.765
Lower back	2.4 (2.5)	2.6 (2.7)	0.489	2.9 (3.1)	2.2 (2.8)	0.027	0.076

All analysis is done as ITT. P-values after test 1–3 show results within the two groups over time and are done using Pearson´s x² test and Student´s *t*-test. P-value to the right shows the Intervention group* Test round effect. ANCOVA analysis is performed. Due to missing data, the number of responders on the different measures varies from 37–54 in intervention and 24–44 in reference group.

### Questionnaire

A questionnaire was completed three times, approximately one week before each test round. The questionnaire was developed for use in all workplaces participating in the FINALE program and consisted of 140 questions mainly of standardized and validated scales [15]. In this paper, responses to questions on musculoskeletal disorders are reported. *Musculoskeletal disorders* were measured with the Nordic questionnaire of musculoskeletal disorders [29], supplemented with questions about localized pain intensity [30].

### Statistical analyses

A power calculation was carried out based on weight change to ensure a copious amount of participants in the intervention and the reference group [14]. Power was set to 0.8 with a significant level of 0.05. At least 30 participants in each group were needed to detect a difference in weight loss of at least 3 kg. With an estimated 30% drop out, 43 participants were needed in each group. Differences between intervention and reference group at baseline were tested with Pearson´s x² for distribution in sex, education (health care workers), current smoking status and the dichotomized parameter for musculoskeletal symptoms in neck, shoulders, upper- and lower back. All other parameters were tested with a Student´s *t*-test. When comparing intervention and reference groups over time, ANCOVA analysis were performed in accordance to the intention-to-treat principle, i.e. all randomized participants are included in the analyses with missing values substituted with carried forward or backwards measured variables. Clusters, age and the investigated value at baseline were included as covariates. All results are given as mean (SD). P < 0.05 are considered statistically significant.

## Results

### Study population

**Figure 2:**
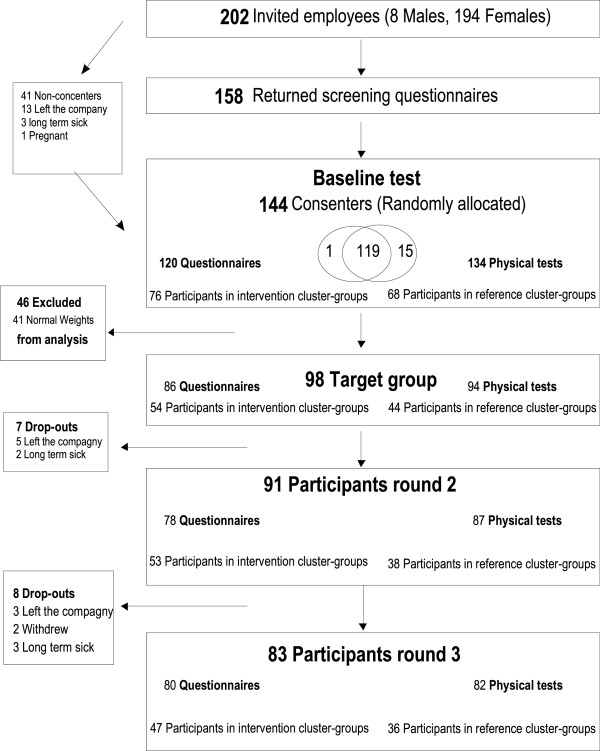
Flow of the participants.

A flowchart of the project is presented in Figure 2. From the employee list, 202 persons (8 men and 194 women), working at least 15 hours/week were invited to participate in the study. Among those, 144 consented to participate and were invited for baseline test and randomly allocated to either the intervention or the reference group. 98 met the full criteria to enter the target group, i.e. women, overweight based on BMI or fat percentage, health care workers or having similar education with daily patient care. After three months 91 were still participating and invited for test 2. At test 3 after 12 months, 83 completed the test.

### Baseline characteristics

Table 1 presents data from baseline (test 1) to 12 months (test 3) in both the intervention and the reference group. As a mean for all participants BMI was 30.6, aerobic fitness 26.3 mL O_2_/kg/min, waist circumference 100.3 and blood pressure 131.8/83.7. No differences between the intervention group and the reference group were found at baseline.

### Changes after 12 months intervention

In the intention to treat analysis, a highly significant Intervention group* Test round effect between the two groups was found for change in weight, BMI and fat percentages from test 1 to test 3. In the intervention group, body weight decreased from 84.2 to 78.4 kg, corresponding to a decrease in BMI from 30.7 to 28.5 and reduced fat percentage from 41.2 to 38.4%.

**Figure 3:**
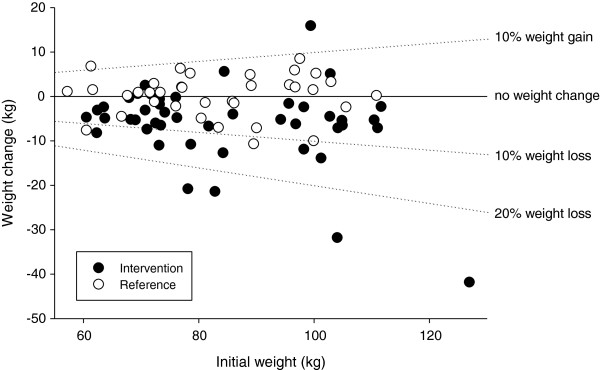
**Weight changes.** Data points represent 12 month body weight change plotted against initial body weight in all individuals who had weight measurements performed in both baseline and 12 month tests. Intervention group n =45, Reference group n = 35.

There was a substantial variation in the weight loss between individuals as shown in Figure 3. In the intervention group the body weight changes over 12 months ranged from +15 to −42 kg, but the majority of the participants had weight losses between 0 and 10 kg. In order to test whether the weight loss depended on initial weight, a linear regression analysis was performed. In the intervention group a weak but significant linear correlation was found between weight change and initial weight (r = 0.30, P < 0.05). However this correlation relied on one outlier with a weight loss of 42 kg and an initial weight of 127 kg, when this individual was removed from the analysis there was no longer a significant correlation between weight loss and initial weight.

From test 1 to test 3, a significant Intervention group* Test round effect was also found for hip and waist circumference, but not for H/W-ratio.

Although there was a significant decrease over time in blood pressure (systolic BP from 134.1 to 125.5 mmHg and diastolic BP from 85.5 to 81.7 mmHg) within the intervention group, the change blood pressure was not different from the reference group (Intervention group* Test round, P > 0.05).

Regarding physical capacity measures, there were no significant Intervention group* Test round effects. Both the reference and the intervention group increased significantly over time in isometric muscle strength, for shoulder elevation and abduction and back extension. Only the intervention group significantly increased aerobic fitness over time, from 25.9 to 27.5 ml/min/kg. Regarding musculoskeletal pain, there was no significant Intervention group* Test round effect. In the intervention group, significant decreased pain level was found for both the neck and right shoulder. Except for the lower back, no changes in pain level in any of the body regions in the reference group were found.

### Fractions of the population in high risk groups based on existing guidelines for BMI, waist circumference and resting blood pressure

Some of the factors that affect the risks for cardiovascular diseases are BMI, waist circumference and blood pressure. In both the intervention and the reference group, subjects in test 1and 3 were classified as above or below the recommended level for BMI (>24.9), waist circumference (>88 cm) and blood pressure (>139/89) in order to define the high risk fraction of the groups. The high risk group based on BMI, waist circumference and blood pressure showed a substantial decrease in the intervention group, in percentages from 90.7, 77.8 and 44.2 in test 1 to 64.8, 66.7 and 27.9 in test 3, respectively. The high risk fractions likewise decreased in the reference group but with much smaller changes.

## Discussion

Twelve months of intervention consisting of diet, physical exercise and cognitive behavioral training significantly decreased body weight, BMI, body fat percentage as well as hip and waist ratio compared to the reference group. However, there were no significant differences between the two groups over time in blood pressure and physical capacity. The interpretation and implications of these findings are discussed below. The findings were based on intention to treat analysis and the workplace intervention was completed by a large proportion (85%) of the targeted overweight female health care workers.

The workplace is suggested to be an efficient arena for weight loss interventions, because workplaces often represent a clustering of different socioeconomic groups and health-related conditions, like overweight and obesity. The present study supports the workplace may be an efficient approach to reach a high-risk group since 93% of the eligible employees were overweight or obese. Using the workplace as a platform for weight loss programs may promote a team spirit among the employees facilitating a sustained effort and long term weight loss [31,32]. The participants tended to form groups at workplaces, often based on gender, educational backgrounds and interests, which makes group-based cognitive behavioral training easier. Moreover, the employees met on a daily basis during the intervention period, and tended to share meals and have opportunities to meet immediately after work for physical exercise or other weight reduction activities [33]. The relatively low drop-out in our study compared to other studies [34,35] supports that the abovementioned factors have strengthened the completion rate in our study-group and ought to be considered in future studies and workplace initiatives for reducing excessive overweight.

The observed decrease of about 6 kg in body weight, of more than 2 units in BMI and with almost 3 percent in body fat among the female overweight health care workers are in the high end of the range of results from previously reported worksite interventions [2]. There were variations in the weight loss, but a substantial proportion of individuals completing the intervention had a weight loss of more than 10% of initial body weight (Figure 3). These findings support that clinically relevant decreases in body weight may be attained with life-style interventions in an occupational setting. If life-style interventions were initiated at more workplaces, hopefully the prevalence of diseases such as obesity, diabetes and cardiovascular diseases would decrease in the future.

The present study design followed the recommendations from recent reviews of integrating diet, physical exercise and cognitive behavioral approaches in order to increase the probability of prolonged weight loss [4,36]. The positive findings of this study after 12 months supports that the integration of these three initiatives seems to be effective for long-term weight loss. Although not tested in this study, the timing and adaptive focus on these three initiatives for long term weight loss may be of importance. The main aim of the intervention after the primary focus on diet in the initial part (3 months) was to maintain the weight loss. We previously reported that that weight loss after the initial three months of the intervention was 3.6 kg on average [14]. However, participants continued on average to lose weight over the following 9 months. This observation supports the potential effectiveness in physical exercise and cognitive behavioral approaches for maintaining and even increasing weight loss. It is however, also possible, that the participants in the intervention group continued to use the dietary tools they had learned in the first three months of the intervention to achieve further weight loss, despite the encouragement to focus on weight maintenance alone.

With respect to physical exercise, our results show no differences in physical capacity (strength and aerobic fitness) between the intervention and reference groups. The main purpose of the physical activity was in our study to help reducing excessive body weight and maintain muscle mass and not specifically to increase physical capacity measures. The physical exercise was therefore not primarily designed for improving specific physical capacities, but merely for promoting energy expenditure in a varied and motivating manner. If the aim was to enhance VO2max, the physical exercise would contain more high intensive activities. This may be the main cause for the lack of effect on physical capacity even though muscle mass was maintained. Maintaining muscle mass has in Henriksen et al. 2012 been pointed out as a specifically important aspect of a successful weight loss program [37]. Also the relative aerobic power in the present study did increase due to the lower body weight and the maintained absolute aerobic capacity. Regarding aerobic power the present results are in accordance with Atlantis E, 2006, although that study suffered from a large drop out and a generally low compliance partly explaining the limited effect on both BMI and physical capacity [38].

After three months intervention, we found a significant larger decrease in blood pressure in the intervention group compared to the reference group [14]. After 12 months, this difference was no longer significant. Many other studies have found blood pressure reductions following life-style based work-place interventions [36,39]. The fact that we did not find this effect after 12 month suggests that the intensity or the compliance of the intervention was not strong enough with respect to physical activity and perhaps further measures should be taken to ensure progression and compliance in the physical training. It should be noted, however, that the blood pressure did decrease compared to baseline within the intervention group, but a concomitant non-significant decrease in the reference group precluded the difference between groups from being statistically significant.

### Strengths and limitations

This one year study was conducted as a cluster randomized single-blinded controlled trial, with data undergoing an intention-to-treat analysis (ITT). In spite of this rather conservative approach, we were able to reveal significant effects on the primary and secondary outcomes. The adherence rate of the study was very high, with a drop out of only 15% after 12 months. A limitation in the study is that in the integrated multiple interventions, the importance of each of the elements cannot be evaluated. Accordingly, we have not assessed the exact adherence to the diet or the other elements in the intervention. The diet was individually adjusted so participants were instructed that they could have a higher intake than calculated if they felt the need and could exercise more in order to keep the aimed calorie deficit. Even though the cognitive part of the sessions closely followed protocol, we could not entirely control what participants brought up for question and therefore we cannot state, that all participants got the precisely same guidance, which of course may affect the outcome. A well-defined protocol was followed concerning the brief physical exercises during sessions, but there was a limitation in the lack of quantitative registration of physical training doses in leisure time. The training logbook was primarily used to facilitate the individual coaching and serve as a motivating factor and no useful quantitative data on physical training could be obtained from these records.

Significant reductions in systolic blood pressure after 12 months were found for both groups, and therefore no significant Intervention group* Test round effects was found. This may be caused by an inability to totally prevent contamination between the groups by the cluster randomization. Finally, the target group only consists of females and the results cannot be extrapolated to males. Concerning statistics, the power calculation reported in the clinical trial and therefore in this paper do not take into account clusters as a covariate in the statistical analyses. However, because of the very clear finding on body weight, we don’t see this as an important limitation for the interpretation of the results. The statistical power is an issue though, with respect to the secondary outcomes in this study. Several ANCOVA models were carried out for testing effects of the intervention on multiple outcomes. The risk for a chance finding may therefore be present. However, reducing the level of significance would substantially increase the risk for a type II error. This aspect ought to be considered when interpreting the results.

The study was not designed for being very cost effective, but to investigate if it’s possible to generate a long-term weight loss among overweight health care workers in a workplace setting. After finding such positive results, a next step is to generate a cost-effective study with the same aim. Therefore, we did not perform a cost-effectiveness evaluation of this study.

## Conclusions

The 12-months workplace intervention among female overweight health care workers consisting of diet, physical exercise and cognitive behavioral training resulted in an average weight loss of about 6 kg, BMI of more than 2 units and body fat with almost 3 percent in an intention to treat analysis. This study shows that an integrated life-style intervention is effective for attaining prolonged weight loss among overweight and obese female workers. Furthermore, the positive results support that workplaces are efficient arenas for weight loss programs among overweight persons.

## Competing interests

The authors declare that they have no competing interests.

## Authors’ contributions

JRC, KS, KO and AHO designed and concepted the study. JRC was responsible for designing the combined intervention and the two parts - the diet and the physical exercise protocol. The cognitive behavioral training and the testing protocol was designed by researchers from National Research Centre for the Working Environment in Copenhagen, Denmark (NRCWE) together with JRC. Concerning the practical part of the study, JRC was responsible for the recruitment of workplaces and participations, the 12 months intervention, the collection of data, statistical analyses and together with IGC performed the data processing. All authors were involved in data interpretation. JRC wrote the first draft, and all authors read and approved the final manuscript.

## Pre-publication history

The pre-publication history for this paper can be accessed here:

http://www.biomedcentral.com/1471-2458/12/625/prepub

## References

[B1] Pohjonen T Age-related physical fitness and the predictive values of fitness tests for work ability in home care work J Occup Environ Med 2001 43 723 730 10.1097/00043764-200108000-00011 11515256

[B2] Anderson LM Quinn TA Glanz K Ramirez G Kahwati LC Johnson DB The effectiveness of worksite nutrition and physical activity interventions for controlling employee overweight and obesity: a systematic review Am J Prev Med 2009 37 340 357 10.1016/j.amepre.2009.07.003 19765507

[B3] Kautiainen S Koivisto AM Koivusilta L Lintonen T Virtanen SM Rimpela A Sociodemographic factors and a secular trend of adolescent overweight in Finland Int J Pediatr Obes 2009 4 360 370 10.3109/17477160902811173 19922053

[B4] Dalle GR Calugi S Centis E El GM Marchesini G Cognitive-behavioral strategies to increase the adherence to exercise in the management of obesity J Obes 2011 201 1 348293 10.1155/2011/348293 PMC2968119 21052533

[B5] Franz MJ VanWormer JJ Crain AL Boucher JL Histon T Caplan W Weight-loss outcomes: a systematic review and meta-analysis of weight-loss clinical trials with a minimum 1-year follow-up J Am Diet Assoc 2007 107 1755 1767 10.1016/j.jada.2007.07.017 17904936

[B6] Blair SN Church TS The fitness, obesity, and health equation: is physical activity the common denominator? JAMA 2004 292 1232 1234 10.1001/jama.292.10.1232 15353537

[B7] Han TS Schouten JS Lean ME Seidell JC The prevalence of low back pain and associations with body fatness, fat distribution and height Int J Obes Relat Metab Disord 1997 21 600 607 10.1038/sj.ijo.0800448 9226492

[B8] van Duijvenbode DC Hoozemans MJ van Poppel MN Proper KI The relationship between overweight and obesity, and sick leave: a systematic review 3 Int J Obes (Lond) 2009 33 807 816 10.1038/ijo.2009.121 19528969

[B9] Cash SW Beresford SA Henderson JA McTiernan A Xiao L Wang CY Dietary and physical activity behaviours related to obesity-specific quality of life and work productivity: baseline results from a worksite trial 1 Br J Nutr 2011 1 9 10.1017/S0007114511006258 PMC3359391 22142517

[B10] van den Berg TI Elders LA de Zwart BC Burdorf A The effects of work-related and individual factors on the Work Ability Index: a systematic review Occup Environ Med 2009 66 211 220 19017690 10.1136/oem.2008.039883

[B11] Miyatake N Miyachi M Tabata I Sakano N Hirao T Numata T Realtionship between muscle strength and anthropometric, body composition parameters in Japanese adolescents Health 2012 4 1

[B12] Winkler A Hebestrelt A Ahrens W Körperliche aktivität und adipositas (Physical activity and obesity) Bundesgesundheitblatt 2011 55 24 34 10.1007/s00103-011-1386-y 22286247

[B13] Capodaglio P Castelnuovo G Brunani A Vismara L Villa V Capodaglio EM Functional limitations and occupational issues in obesity: a review Int J Occup Saf Ergon 2010 16 507 523 21144269 10.1080/10803548.2010.11076863

[B14] Christensen JR Faber A Ekner D Overgaard K Holtermann A Sogaard K Diet, physical exercise and cognitive behavioral training as a combined workplace based intervention to reduce body weight and increase physical capacity in health care workers - a randomized controlled trial BMC Public Health 2011 11 671 10.1186/1471-2458-11-671 21871113 PMC3175468

[B15] Holtermann A Jorgensen MB Gram B Christensen JR Faber A Overgaard K Worksite interventions for preventing physical deterioration among employees in job-groups with high physical work demands: background, design and conceptual model of FINALE BMC Public Health 2010 10 120 10.1186/1471-2458-10-120 20214807 PMC2841104

[B16] Basiotis PP Welsh SO Cronin FJ Kelsay JL Mertz W Number of days of food intake records required to estimate individual and group nutrient intakes with defined confidence J Nutr 1987 117 1638 1641 3655942 10.1093/jn/117.9.1638

[B17] Azadbakht L Surkan PJ Esmaillzadeh A Willett WC The Dietary Approaches to Stop Hypertension eating plan affects C-reactive protein, coagulation abnormalities, and hepatic function tests among type 2 diabetic patients J Nutr 2011 141 1083 1088 10.3945/jn.110.136739 21525259 PMC3137257

[B18] Nordic Nutrition Recommendation 2004 Norden 2004 1 436

[B19] Seagle HM Strain GW Makris A Reeves RS American Dietetic Association Position of the American Dietetic Association: weight management 2009 109 2 330 46 10.1016/j.jada.2008.11.041 19244669

[B20] Linton SJ Bradley LA Jensen I Spangfort E Sundell L The secondary prevention of low back pain: a controlled study with follow-up Pain 1989 36 197 207 10.1016/0304-3959(89)90024-9 2521930

[B21] Linton SJ Andersson T Can chronic disability be prevented? A randomized trial of a cognitive-behavior intervention and two forms of information for patients with spinal pain Spine (Phila Pa 1976) 2000 25 2825 2831 10.1097/00007632-200011010-00017 11064530

[B22] Appleyard M Hansen T Schnohr P Jensen G Nyboe J The Copenhagen City Heart Study: Østerbroundersøgelsen: a book of tables with data from the first examination (1976-78) and a five year follow-up (1981-83) Scand J Soc Med 1989 170 suppl 41 1 160 2711133

[B23] Andersen LB A maximal cycle exercise protocol to predict maximal oxygen uptake Scand J Med Sci Sports 1995 5 143 146 7552756 10.1111/j.1600-0838.1995.tb00027.x

[B24] Essendrop M Maul I Laubli T Riihimaki H Schibye B Measures of low back function: a review of reproducibility studies Clin Biomech (Bristol, Avon) 2002 17 235 249 10.1016/S0268-0033(02)00022-0 12034116

[B25] Schibye B Hansen AF Sogaard K Christensen H Aerobic power and muscle strength among young and elderly workers with and without physically demanding work tasks Appl Ergon 2001 32 425 431 10.1016/S0003-6870(01)00034-5 11534787

[B26] Fairfax AH Balnave R Adams RD Variability of grip strength during isometric contraction Ergonomics 1995 38 1819 1830 10.1080/00140139508925229 7671859

[B27] Backman E Johansson V Hager B Sjoblom P Henriksson KG Isometric muscle strength and muscular endurance in normal persons aged between 17 and 70 years Scand J Rehabil Med 1995 27 109 117 7569820

[B28] Yates JW Kamon E Rodgers SH Champney PC Static lifting strength and maximal isometric voluntary contractions of back, arm and shoulder muscles Ergonomics 1980 23 37 47 10.1080/00140138008924716 7363885

[B29] Kuorinka I Jonsson B Kilbom A Vinterberg H Biering-Sorensen F Andersson G Standardised Nordic questionnaires for the analysis of musculoskeletal symptoms Appl Ergon 1987 18 233 237 10.1016/0003-6870(87)90010-X 15676628

[B30] Videman T Nurminen T Tola S Kuorinka I Vanharanta H Troup JD Low-back pain in nurses and some loading factors of work Spine (Phila Pa 1976) 1984 9 400 404 10.1097/00007632-198405000-00013 6236565

[B31] Brownell KD Cohen RY Stunkard AJ Felix MR Cooley NB Weight loss competitions at the work site: impact on weight, morale and cost-effectiveness Am J Public Health 1984 74 1283 1285 10.2105/AJPH.74.11.1283 6437259 PMC1652048

[B32] Peregrin T Weighing in on corporate wellness programs and their impact on obesity J Am Diet Assoc 2005 105 1192 1194 10.1016/j.jada.2005.06.012 16182630

[B33] Rigsby A Gropper DM Gropper SS Success of women in a worksite weight loss program: Does being part of a group help? Eat Behav 2009 10 128 130 10.1016/j.eatbeh.2009.01.002 19447356

[B34] Malone M Alger-Mayer SA Anderson DA The lifestyle challenge program: a multidisciplinary approach to weight management Ann Pharmacother 2005 39 2015 2020 10.1345/aph.1G287 16288070

[B35] Benedict MA Arterburn D Worksite-based weight loss programs: a systematic review of recent literature Am J Health Promot 2008 22 408 416 10.4278/ajhp.22.6.408 18677881

[B36] Pescatello LS Murphy D Vollono J Lynch E Bernene J Costanzo D The cardiovascular health impact of an incentive worksite health promotion program Am J Health Promot 2001 16 16 20 10.4278/0890-1171-16.1.16 11575051

[B37] Henriksen M Christensen R Danneskiold-Samsoe B Bliddal H Changes in lower extremity muscle mass and muscle strength after weight loss in obese patients with knee osteoarthritis: a prospective cohort study Arthritis Rheum 2012 64 438 442 10.1002/art.33394 22161649

[B38] Atlantis E Chow CM Kirby A Fiatarone Singh MA Worksite intervention effects on physical health: a randomized controlled trial Health Promot Int 2006 21 191 200 10.1093/heapro/dal012 16595619

[B39] Shimizu T Horiguchi I Kato T Nagata S Relationship between an interview-based health promotion program and cardiovascular risk factors at Japanese companies J Occup Health 2004 46 205 212 10.1539/joh.46.205 15215662

